# Risk factors for race-day fatality in flat racing Thoroughbreds in Great Britain (2000 to 2013)

**DOI:** 10.1371/journal.pone.0194299

**Published:** 2018-03-21

**Authors:** Sarah M. Rosanowski, Yu-Mei Chang, Anthony J. Stirk, Kristien L. P. Verheyen

**Affiliations:** 1 Veterinary Epidemiology, Economics and Public Health Group, Department of Pathobiology and Population Sciences, The Royal Veterinary College, University of London, North Mymms, Hatfield, Hertfordshire, United Kingdom; 2 Research Office, The Royal Veterinary College, University of London, London,United Kingdom; 3 British Horseracing Authority, London, United Kingdom; Texas A&M University College Station, UNITED STATES

## Abstract

A key focus of the racing industry is to reduce the number of race-day events where horses die suddenly or are euthanased due to catastrophic injury. The objective of this study was therefore to determine risk factors for race-day fatalities in Thoroughbred racehorses, using a cohort of all horses participating in flat racing in Great Britain between 2000 and 2013. Horse-, race- and course-level data were collected and combined with all race-day fatalities, recorded by racecourse veterinarians in a central database. Associations between exposure variables and fatality were assessed using logistic regression analyses for (1) all starts in the dataset and (2) starts made on turf surfaces only. There were 806,764 starts in total, of which 548,571 were on turf surfaces. A total of 610 fatalities were recorded; 377 (61.8%) on turf. In both regression models, increased firmness of the going, increasing racing distance, increasing average horse performance, first year of racing and wearing eye cover for the first time all increased the odds of fatality. Generally, the odds of fatality also increased with increasing horse age whereas increasing number of previous starts reduced fatality odds. In the ‘all starts’ model, horses racing in an auction race were at 1.46 (95% confidence interval (CI) 1.06–2.01) times the odds of fatality compared with horses not racing in this race type. In the turf starts model, horses racing in Group 1 races were at 3.19 (95% CI 1.71–5.93) times the odds of fatality compared with horses not racing in this race type. Identification of novel risk factors including wearing eye cover and race type will help to inform strategies to further reduce the rate of fatality in flat racing horses, enhancing horse and jockey welfare and safety.

## Introduction

Fatalities on race-day due to sudden death in apparently healthy horses or catastrophic injury resulting in euthanasia are a concern for a racing industry seeking to maintain racing safety and enhance racehorse welfare. In Great Britain (GB), the incidence of race-day fatality during flat racing has been reported at between 0.76 and 0.9 per 1000 flat racing starts [1–3]. Most race-day fatalities are due to musculoskeletal injuries [2–6], with the incidence of sudden death reported at between 0.07 and 0.09 per 1000 flat racing starts [3, 4, 7].

One way to improve the safety of racing for both horses and jockeys is by identifying risk factors for race-day fatality, to enable the racing industry to implement effective change. Previous studies investigating both jump and flat racing fatalities have identified that the risk of fatality increased with increasing age at first race for all-cause fatalities [2] and for fatality due to lateral condylar fractures of the third metacarpal or third metatarsal bones [8]. Further, it was reported that two-year-old horses and horses older than five years old were at a higher risk of fatal fracture than horses aged three to five years [9]. Other studies have identified associations between race-day fatality and male horses, prior racing intensity, racing on an all-weather surface, racing on firmer going, longer racing distances, season the race was held and racing over jumps [2, 4, 6, 7, 10–13].

No recent studies have specifically focussed on risk factors for flat racing fatality. Consequently, the objective of the current study was to determine risk factors for fatality in Thoroughbred racehorses participating in flat racing in Great Britain. Identifying modifiable risk factors will enable the racing industry to implement effective changes to enhance horse and jockey welfare and safety.

## Methods

Data included all Thoroughbred flat racing starts in GB between 1^st^ January 2000 and 31^st^ December 2013, excluding National Hunt flat racing starts. The study population consisted of all horses that raced, as defined by entering the starting stalls prior to racing, during the study period. Information regarding all starts, including horse demographics, race and course information was obtained from the Weatherbys racing database (www.weatherbys.co.uk). In addition, the British Horseracing Authority (BHA) provided data on all race-day injuries and fatalities recorded in the BHA’s injury database by on-course veterinarians. The racing and injury databases were merged by matching on horse and race identification numbers, creating a single custom-designed SQL database.

The outcome of fatality was defined as an event that resulted in the death or euthanasia of the horse on race-day and included fatality from all causes. Fatalities were recorded by official racecourse veterinarians and the reported reason(s) for fatality were primarily based on clinical examination and presumptive diagnosis, generally without further diagnostic investigations or post mortem examination.

The unit of interest for the study was at the start level and one horse could have multiple starts during the study period. Exposure variables were sourced from the racing dataset and additional performance variables were created using data prior to the current start. These included the number of starts, average score (30 for a win, 20 for a place, 10 for a completed run, 0 for a start but failed to finish) [14], percentage wins, percentage placed and percentage of failures to finish. These measures were calculated for each horse, trainer and jockey for all race starts (including National Hunt racing starts) and all flat racing starts. The number of starts per horse in the previous 15 and 30 days were calculated. The number of days since last start, defined as racing intensity, was categorised as five groups: first start, start in the previous 1 to 7 days, start in the previous 8 to 93 days, start in the previous 94 to 364 days and last start more than 1 year ago. Age was described as current age (in years: 2, 3, 4, 5, 6 and 7+) and age at first start (in years: 2, 3, 4+). In addition, a binary variable of first year racing (yes/no) was created. Sex was categorised in three groups: stallions, colts and rigs, geldings, and mares and fillies. The official track rating or condition, called going, was categorised in five levels, from hard, firm or fast to soft, heavy or slow (see Table 1). Eye cover was defined as any type of covering around the eyes allowed during racing under official race rules: eye shields, blinkers or visors. For each start, horses were categorised as not wearing eye cover, wearing eye cover for the first time or wearing eye cover (not for the first time).

**Table 1:** All cause fatality stratified by musculoskeletal injury (MSI) or non-musculoskeletal injury (non-MSI) and racecourse surface.

Cause of fatality	All-weather surface fatalities Number (%)	Turf surface fatalities Number (%)
**MSI (total)**	207 (88.8)	327 (86.7)
**Fracture**	176 (75.5)	280 (74.3)
**Dislocation (fetlock)**	6 (2.6)	7 (1.9)
**Tendon or ligament injury**	19 (8.2)	33 (8.8)
**Multiple MSI**	6 (2.6)	6 (1.6)
**MSI other**	0 (0)	1 (0.3)
**MSI + non-MSI (total)**	2 (0.9)	1 (0.3)
**Non-MSI (total)**	24 (10.3)	49 (13.0)
**Vascular catastrophe**	20 (8.6)	39 (10.3)
**Epistaxis**	2 (0.9)	3 (0.8)
**Laceration**	1 (0.4)	2 (0.5)
**Multiple non-MSI**	1 (0.4)	0 (0)
**Non-MSI other**	0 (0)	5 (1.3)

### Statistical analysis

Multivariable logistic regression was used to determine explanatory variables that were associated with a fatal outcome. A first analysis included all race starts in the dataset, on both turf and all-weather surfaces. An additional analysis investigated risk factors for fatality on turf surfaces only. Exposure variables were screened using univariable logistic regression and those with a likelihood ratio test P-value <0.25 were selected for inclusion in a multivariable model. A preliminary multivariable model was built using a manual backwards method of elimination in which variables were retained in the model if the likelihood ratio test P-value was <0.05 and/or by comparing Akaike Information Criteria (AIC). The likelihood ratio test was used as the primary selection criterion, with AIC used when variables were similar, for example modelled as continuous or categorical, or correlated. Linearity was assessed for continuous predictors and if non-linearity was identified, either quadratic or fractional polynomial terms were included in the model or the variable was categorised based on quartiles.

Potential two-way interaction terms were identified using a classification tree method [15]. Briefly, all variables selected for inclusion in the multivariable model development (P<0.25) were assessed for interaction by randomly dividing all non-fatal starts into data subsets, with the size of subset calculated as the nearest integer of non-fatal starts/(fatal starts *2). Each subset was combined with the fatal starts observations and a classification tree generated for each subset. In each classification tree analysis, each time the tree branched, a two-way interaction term was identified. The default control parameters were used for building each classification tree, so no extra pruning or stopping rules were applied [16]. Interactions identified in at least 10% of the trees were assessed in the multivariable mixed effects logistic model and retained if the likelihood ratio P-value was <0.05.

A mixed effects logistic regression model was used to account for multiple starts by a horse. In addition, individual random effect terms were added to the final (fixed effects) models to account for the clustering of starts by trainer, jockey, race, race meeting, course, sire and dam. Due to computational constraints, these were added to the model individually and separately from the random effect term accounting for repeated measures by horse.

The fitted probability of fatality was calculated based on the final mixed effects multivariable models. Residual values (observed outcome minus the fitted probability) were calculated to assess model fit; in a well-fitting model, residuals would be near zero. R version 3.2.2 R core [17] and the package rpart [16] were used for the classification tree model. Stata version 13 was used for the mixed effects logistic regression modelling [18].

## Results

### Risk factors for fatality–all flat racing starts

During the study period there were 806,764 starts and 610 fatalities, resulting in a fatality incidence of 0.76 per 1000 starts. Causes of fatality are summarised in Table 1. Descriptive statistics for the continuous variables included in the multivariable models are presented in Table 2. The univariable model for fatality using all race starts is presented in S1 Table, and the multivariable model in Table 3. The between-horse variation was not significant in the model (P = 0.44; n = 67,582). Of the other random effects terms, only trainer was significant (P = 0.01; n = 1362) and therefore included in the final model. There were no changes (>5%) in the odds ratios or P-values between the fixed effects-only and any of the random effects models.

**Table 2:** Median (interquartile range) of the continuous variables included in the multivariable logistic regression models for all starts, and turf only starts, for Thoroughbreds racing in Great Britain, 2000 to 2013.

Variable	All starts (n = 806,764)	Fatal starts (n = 610)	All turf starts (n = 548,571)	Turf fatal starts(n = 377)
**Race-level variables**				
**Distance (metres)**	1400 (1200–2000)	1600 (1400–2400)	1400 (1200–2000)	1600 (1200–2200)
**Horse-level variables**				
**Number of horse starts**	9 (4–20)	8 (4–18)	6 (3–14)	6 (3–13)
**Horse average performance score**	13.70 (10–15.59)	13.71 (10.00–16.15)	13.33 (10.00–15.71)	13.33 (10.00–16.54)

**Table 3:** Multivariable logistic regression results of risk factors for race-day fatality in British flat racing Thoroughbreds (2000 to 2013), including a random effect for trainer (P = 0.01).

Variable	Level	No. of Cases	No. of Starts	Incidence/ 1000 starts	Odds Ratio (95% Confidence Interval)	Wald P value	Likelihood Ratio P Value
Course-level variables							
Surface	Turf	377	548,571	0.69	1		0.001
	All-weather	233	258,193	0.90	1.52 (1.19–1.95)	0.001	
Going	Hard or Firm	25	29,294	0.85	1.40 (0.90–2.16)	0.13	0.006
	Good to firm, Standard to fast	179	220,379	0.81	1.29 (1.02–1.63)	0.04	
	Good, Standard	317	394,225	0.80	1		
	Good to soft, Standard to slow	46	84,371	0.55	0.81 (0.58–1.14)	0.23	
	Soft, heavy or slow	43	78,495	0.55	0.80 (0.56–1.14)	0.21	
Race-level variables							
Distance (per 100 metres)					1.05 (1.03–1.06)	<0.001	<0.001
Year	2000	31	48,932	0.63	0.65 (0.41–1.02)	0.06	0.03
	2001	38	53,239	0.71	0.77 (0.50–1.17)	0.22	
	2002	58	52,999	1.09	1.24 (0.85–1.82)	0.26	
	2003	30	52,919	0.57	0.64 (0.41–1.01)	0.06	
	2004	44	58,778	0.75	0.91 (0.61–1.37)	0.66	
	2005	40	60,090	0.67	0.81 (0.54–1.23)	0.33	
	2006	32	59,242	0.54	0.63 (0.41–0.99)	0.04	
	2007	42	60,078	0.70	0.82 (0.54–1.23)	0.33	
	2008	44	61,998	0.71	0.83 (0.56–1.25)	0.38	
	2009	51	61,968	0.82	0.94 (0.64–1.39)	0.76	
	2010	58	60,816	0.95	1.10 (0.75–1.60)	0.62	
	2011	38	59,592	0.64	0.73 (0.48–1.12)	0.15	
	2012	53	58,593	0.90	1.07 (0.73–1.57)	0.73	
	2013	51	57,520	0.89	1		
Season	Spring	120	186,632	0.64	1		0.01
	Summer	240	311,753	0.77	1.25 (0.99–1.57)	0.05	
	Autumn	176	207,788	0.85	1.36 (1.07–1.74)	0.01	
	Winter	74	100,591	0.74	0.89 (0.65–1.23)	0.48	
Auction race	No	560	753,179	0.74	1		0.03
	Yes	50	53,585	0.93	1.46 (1.06–2.01)	0.02	
Horse-level variables							
Current age (years)	2	101	155,367	0.65	1		<0.001
	3	186	254,906	0.73	1.73 (1.27–2.34)	<0.001	
	4	98	154,099	0.64	1.96 (1.33–2.89)	0.001	
	5	81	94,093	0.86	3.06 (2.02–4.64)	<0.001	
	6	49	60,662	0.81	3.24 (2.03–5.19)	<0.001	
	7+	95	87,637	1.08	5.11 (3.24–8.05)	<0.001	
First year racing	No	413	583,391	0.71	1		<0.001
	Yes	197	223,373	0.88	2.16 (1.66–2.80)	<0.001	
Number of previous flat starts					0.98 (0.98–0.99)	<0.001	<0.001
Eye cover	No	517	701,796	0.74	1		0.01
	Yes, first time	28	20,654	1.36	1.83 (1.25–2.69)	<0.001	
	Yes, worn previously	65	84,314	0.77	1.18 (0.90–1.54)	0.23	
Horse average performance score					1.02 (1.005–1.04)	0.01	0.01

In total, 281 trainers (20.6%) had horses that experienced a race-day fatality: 52.0% of these (n = 146) had one fatality, 23.1% (n = 65) had two fatalities and 16.4% (n = 46) had three or four fatalities. Three trainers had ten or more fatalities over the 14-year study period. Horses racing on an all-weather surface, increased firmness of the going, increasing race distance, races held in summer and autumn, auction races and increasing horse average performance score all increased the odds of fatality. Horses that were wearing eye cover for the first time were at 1.8 times the odds of fatality, compared with horses that did not wear eye cover. As age increased, the odds of fatality increased and horses in their first year of racing were at higher odds of fatality. The odds of fatality varied by year. Increasing number of horse starts on the flat reduced the odds of fatality. No significant interaction terms were identified.

The residual values had a median of 0.9969 (IQR 0.9952 to 0.9978) and -0.0002 (IQR -0.0002 to -0.0001) for fatal and non-fatal starts, respectively.

### Risk factors for fatality–turf flat racing starts only

During the study period there were 548,571 starts and 377 fatalities on turf, resulting in an incidence of 0.69 per 1000 starts. The univariable and multivariable models for fatality on turf surfaces are presented in S2 Table and Table 4, respectively. The between-horse variation was not significant in the model (P = 0.47; n = 62,859) and of the other random effects terms, only trainer was significant (P = 0.03; n = 1218) and therefore included in the final model. There were no changes (>5%) in the odds ratios or P-values between the fixed effects-only and any of the random effects models.

**Table 4:** Multivariable logistic regression results of risk factors for race-day fatality on turf racecourses in British flat racing Thoroughbreds (2000 to 2013), including a random effect for trainer (P = 0.03).

Variable	Level	No. of Cases	No. of Starts	Incidence/1000 starts	Odds Ratio (95% Confidence Interval)	Wald P value	Likelihood Ratio P value
Course-level variables							
Going	Hard or Firm	24	29,002	0.83	1.3 (0.83–2.03)	0.26	0.02
	Good to firm	172	217,424	0.79	1.19 (0.93–1.52)	0.17	
	Good	102	152,511	0.67	1		
	Good to soft	41	76,665	0.53	0.78 (0.54–1.13)	0.19	
	Soft or heavy	38	72,969	0.52	0.76 (0.52–1.11)	0.15	
Race-level variables							
Distance (per 100 metres)					1.05 (1.03–1.07)	<0.001	<0.001
Group 1 race	No	366	543,966	0.67	1		0.002
	Yes	11	4,605	2.39	3.19 (1.71–5.93)	<0.001	
Horse-level variables							
Current age (years)	2	72	119,631	0.60	1		<0.001
	3	129	181,290	0.71	1.76 (1.24–2.51)	0.002	
	4	49	99,277	0.49	1.60 (0.99–2.60)	0.06	
	5	49	58,347	0.84	3.00 (1.82–4.97)	<0.001	
	6	30	37,453	0.80	3.16 (1.78–5.59)	<0.001	
	7+	48	52,573	0.91	4.09 (2.32–7.19)	<0.001	
First year racing	No	238	382,847	0.62	1		<0.001
	Yes	139	165,724	0.84	2.81 (2.04–3.88)	<0.001	
Number of previous horse starts (on turf)					0.98 (0.97–0.995)	0.006	0.004
Eye cover	No	326	490,679	0.66	1		0.01
	Yes, first time	21	19,676	1.07	2.08 (1.33–3.24)	0.001	
	Yes, worn previously	30	38,216	0.79	1.23 (0.84–1.81)	0.29	
Horse average performance score (all flat starts)					1.02 (1.00–1.04)	0.05	0.05
365 days or more since last race start	No	363	540,728	0.67	1		0.04
	Yes	14	7,843	1.79	1.92 (1.10–3.35)	0.02	

In total, 205 trainers (16.8%) had horses that experienced a race-day fatality on turf: 62.0% of these (n = 127) had one fatality, 20.5% (n = 42) had two fatalities and 11.7% (n = 24) had three or four fatalities. One trainer had more than ten fatalities over the 14-year study period. The odds of fatality increased with an increased firmness of the going, increasing race distance, increasing horse average performance, horses in their first year of racing, and wearing eye cover for the first time. Generally, the odds of fatality increased with increasing horse age. Increasing number of horse starts on turf reduced the odds of fatality. Starting in a Group 1 race increased the odds of fatality as did not starting in a race for more than 365 days. No significant interaction terms were identified.

The median residual values were 0.9992 (IQR 0.9988 to 0.9995) and -0.0005 (IQR -0.0008 to -0.0004) for fatal and non-fatal starts, respectively.

## Discussion

This study has identified both previously established and novel risk factors associated with flat racing fatality in GB. Racing on an all-weather surface, increasing firmness of going and increasing racing distance were identified as risk factors, while an increasing number of career starts was protective. These findings were similar to those of previous studies investigating risk factors for flat racing fatality [2, 12] and fatal third metacarpal fracture [19], jump racing superficial digital flexor tendinopathy (SDFT) [14, 20] and fatality and distal limb fracture on all-weather tracks [21]. Whilst similarities have been noted, novel risk factors for fatality including horses wearing eye cover, race type, increasing horse performance and horses in their first year racing were also identified.

Vision restriction, particularly for the first time, was a risk factor in both models. Previous studies have investigated the use of blinkers as a risk factor for race-day fatality [9, 10, 22] or injury [23], but did not identify associations following multivariable modelling. These studies investigated the use of eye cover, but not whether eye cover was a new experience for the horse. The reason for the association with fatality in the current study is unclear although may be due to a change in the horse’s depth perception or ability to judge distance, detect changes in the racing surface or avoid interference by other horses. The risk of fatality was not significantly different in horses who had worn eye cover previously compared to those not wearing eye cover, suggesting that horses may become acclimatised to vision restriction. Alternatively, it could also be that trainers decide to use eye cover in an attempt to enhance performance, with poor performance resulting from subclinical pathology. Such horses would be at higher risk of (fatal) injury, in particular when wearing eye cover for the first time. Further research in this area, particularly investigating when and why eye cover is used in flat racing horses and how eye cover affects the vision and the performance of horses under race conditions is advisable.

Race type was a risk factor for fatality in both models, with horses entered in an auction race in the all-starts model or Group 1 races in the turf model at a higher risk of fatality. Auction races are for two- or three-year-olds that were sold by public auction at specific sales [24]. As such, horses in auction races are generally younger horses that have not previously won a race. Group 1 races are international-level championship races with high purses (prize money to be won) [25]. No Group 1 races have been held on all-weather surfaces, so this factor was not assessed in the ‘all starts’ model. It is unclear why the likelihood of fatality increased for these race types, although it should be noted that horses competing in Group 1 races would be considered the highest quality of racehorse, while horses in auction races less so. As such, horses training for Group 1 races may be trained more intensively than horses competing in auction races. One limitation of the current study was that no training data were collected for horses, so comparison between training regimens is not possible. Despite this, differences in race-day fatality rates between trainers have been identified in the current study. While most trainers with a race-day fatality only had one fatality over the study period, other trainers had more fatalities. This finding and the higher risk identified in Group 1 and auction horses indicates that further investigation into training practices is warranted in order to reduce the rates of race-day fatality.

Regardless of race type, horses with higher average performance scores (i.e. better performing horses) had an increased risk of fatality. In the current study, horse score was based on a simple weighting of wins, placings, and starts where horses did and did not finish, rather than a measure of the quality or value of the horse or race entered. A previous study in North America identified that horses with a higher perceived chance of winning a race (i.e. higher perceived performance), as determined by betting odds, were more likely to experience a fatal event in that race than horses with lower odds of winning a race [26]. In contrast, studies in GB that have used official race ratings as a measure of performance identified horses with lower performance ratings as being more likely to have a race-day fatality [2] or tendon injury [20].

Inexperienced horses in their first year of racing were at a higher risk of fatality, compared with horses more advanced in their racing career. This finding is consistent with studies that have identified increasing bone density and strength in the first year [27] or two years of training [28, 29]. Unlike previous studies, which identified bone remodelling in two- and three-year-old horses in comparison with untrained cohorts, the effect identified in the current study was regardless of age, although most horses in GB do start their racing careers in their second or third year. Additionally, horses that had not raced in the previous 365 days were at a higher risk of fatality than horses that had raced within a shorter time frame. Bone remodelling and repair occurs when a horse is rested after a period of training. Firth, Rogers (29) identified that bone remodelling occurs as soon as 60 days after the cessation of training. The balance between bone modelling, microdamage and repair is important during any stage of a horse’s training, but may require special consideration for horses that are returning after a break in their racing career [30, 31]. Alternatively, it is possible that the reason for not having raced for over a year is related to previous injury or poor performance, with poor performance potentially masking subclinical injury. As such, this finding may demonstrate a ‘healthy horse’ effect, with horses able to race more often more likely to be at a lower risk of injury or fatality as there is no evident clinical or underlying pathology.

The association between horse age and race-day fatality has been identified previously [6, 8, 9, 12, 21], with studies identifying increasing risk with increasing age at which a horse started racing [2, 12, 21, 26]. While age-related variables (age at first start, current age or years in racing) have been included in most race-day risk factor models, few have included variables to account for both the current age of the horse and the age at which they started racing [12, 21]. The current study, rather than providing conflicting evidence regarding the effect of age on the risk of race-day fatality, through the inclusion of first year racing, adds growing evidence regarding the complexity of age as a risk factor for race-day fatality. The importance of career length and the age or stage that horses start racing [8] and that bone microdamage, which can lead to failure, increases with horse age [32] has been noted previously.

Regardless of the firmness of the going or whether the start was held on a turf or an all-weather surface, racing in summer or autumn had the highest risk of fatality. This is similar to previous studies, where racing in summer was identified as increasing the risk of SDFT in jumping horses [14, 20] and increasing the risk of sudden death [7]. It is possible that changes to the ground surface, which may not be accurately measured by the official race going, may occur during the summer and autumn months, increasing the likelihood of fatality. However, this is unlikely to be the complete picture. Firstly, the flat racing season runs from April until early November, with few races held in winter [33]. Thus, the risk of fatality increased with the progression of the racing reason. This could be because trainers may be under more pressure to race previously unraced or lightly-raced horses before the end of the racing season, with such horses being more prone to racing injury because of underlying issues that prevented them from racing earlier on in the season. Additionally, potential seasonal changes in training surfaces and increased time in training and/or training intensity for horses racing later in the flat racing season could play a role. It was not possible to take account of such factors in the current study, as training data are not routinely collected.

Starts in 2000, 2003 and 2006 were less likely to experience a race-day fatality than starts in 2013, although only significantly so for 2006. The reasons for this finding are unclear, although the recording of race-day data changed to an electronic format in 2004 [3], and the recording of fatal events may have improved since that time. Alternatively, variation between years may have occurred by chance, given the large number of categories for this variable.

Although classification tree models have been used as an alternative to logistic regression analyses [15, 34], classification trees were only used in this study to identify potential two-way interaction terms for inclusion in the logistic regression model. When considering interactions associated with a binary outcome, the classification tree technique allows the rapid screening of a large number of continuous or categorical exposure variables with no assumptions regarding distributions [15]. While no interaction terms were identified here, a previous study using the technique did identify two interaction terms associated with fatality on all-weather surfaces in GB: race distance x number of race starts and horse age x age at first start [21]. These interactions may have been missed when only considering potential interactions based on biological plausibility. Only fixed effects were included in the construction of the classification trees and the identification of 2-way interactions, hence the prediction performance of these trees was not assessed. This is mainly due to the difficulty of incorporating the random effects (horse, trainer or jockey) in the classification tree analysis, although a recently developed method has indicated the possibility of incorporating random effects in the simulated study [35]. Whilst this study has accounted for the multifactorial nature of race-day fatality by using repeated measures logistic regression modelling, there are some limitations in the dataset and analysis presented here. Firstly, all-cause fatalities were combined, so the risk factors for fatality were assumed to be the same regardless of whether a horse suffered a catastrophic musculoskeletal injury requiring euthanasia or a vascular catastrophe or sudden death from another cause. This approach was taken as the occurrence of fatality in during the study period was low, and so the study did not have enough power to further dissect out risk factors for specific types of race-day fatality. It might be expected that risk factors for sudden death would differ when compared with fracture, tendon, ligament and joint injuries, risk factors for which in turn would vary. However, previous studies investigating sudden death [7] and fatal distal limb fracture [9] have identified similar risk factors to those presented here, such as increasing horse age and increasing race distance. Secondly, although the residual values indicated adequate model prediction of non-fatal starts, because the incidence of fatality was low, the predictive ability of the model for fatal starts was also low. As such, the current models are unlikely to be useful for predicting the occurrence of future fatalities and further work is needed to enable better identification of individuals at risk of an adverse outcome.

## Conclusions

This study has identified specific and, in some cases, potentially modifiable risk factors for fatality in flat racing in Great Britain. Previously identified risk factors of going, race distance and age, plus novel risk factors of eye cover and race type are important risk factors for the occurrence of race-day fatality in flat racing Thoroughbreds. The results from this study will help to inform intervention strategies aimed at further reducing the rate of fatality in flat racing horses, enhancing horse and jockey welfare and safety.

## Ethical approval

Consent to use and store the data included in this study was obtained from the British Horseracing Authority. The project was ethically reviewed by the Clinical Research Ethical Review Board at the Royal Veterinary College. Ethical approval was granted, approval number URN 2015 1362.

## Supporting information

S1 Table Univariable logistic regression results for race-day fatality in British flat racing Thoroughbreds (2000 to 2013). Values shown for variables with a likelihood P value of <0.25 and screened for inclusion in the multivariable model.
(DOCX)

S2 Table Univariable logistic regression results of risk factors for race-day fatality on turf racecourses in British flat racing Thoroughbreds (2000 to 2013). Values shown for variables with a likelihood P value of <0.25 and screened for inclusion in the multivariable model.
(DOCX)

## Abbreviations

BHA: British Horseracing Authority
GB: Great Britain
OR: Odds Ratio
SDFT: Superficial digital flexor tendinopathy
95% CI: 95% confidence interval
