# Supplementary material for: Risk factors for race-day fatality in flat racing Thoroughbreds in Great Britain (2000 to 2013)
Source: PLoS One. 2018 Mar 21;13(3):e0194299. doi: 10.1371/journal.pone.0194299 (PMC5862470; doi:10.1371/journal.pone.0194299)
Supplement: S1 Table — Values shown for variables with a likelihood P value of <0.25 and screened for inclusion in the multivariable model. (DOCX) [file pone.0194299.s001.docx]

S1 Table: Univariable logistic regression results for race-day fatality in British flat racing Thoroughbreds (2000 to 2013). Values shown for variables with a likelihood P value of <0.25 and screened for inclusion in the multivariable model.

| Variable | Level | No. of Cases | No. of Starts | Incidence /1000 starts | Odds ratio (95% Confidence Interval) | Wald P value | Likelihood ratio P value |
| --- | --- | --- | --- | --- | --- | --- | --- |
| Course-level | |  |  |  |  |  |  |
| Surface | Turf | 377 | 548,571 | 0.69 | 1 |  | 0.001 |
|  | All-weather | 233 | 258,193 | 0.90 | 1.31 (1.12 - 1.55) | 0.001 |  |
|  |  |  |  |  |  |  |  |
| Going | Hard or Firm | 25 | 29,294 | 0.85 | 1.06 (0.71 - 1.60) | 0.77 | 0.01 |
|  | Good to firm, Standard to fast | 179 | 220,379 | 0.81 | 1.01 (0.84 - 1.21) | 0.91 |  |
|  | Good, Standard | 317 | 394,225 | 0.8 | 1 |  |  |
|  | Good to soft, Standard to slow | 46 | 84,371 | 0.55 | 0.68 (0.50 - 0.92) | 0.01 |  |
|  | Soft, heavy or slow | 43 | 78,495 | 0.55 | 0.68 (0.50 - 0.94) | 0.02 |  |
|  |  |  |  |  |  |  |  |
| Race-level | |  |  |  |  |  |  |
| Distance (metres) | |  |  |  | 1.06 (1.05 - 1.07) | <0.001 | <0.001 |
|  |  |  |  |  |  |  |  |
| Year | 2000 | 31 | 48,932 | 0.63 | 0.71 (0.46 - 1.12) | 0.14 | 0.04 |
|  | 2001 | 38 | 53,239 | 0.71 | 0.80 (0.53 - 1.23) | 0.31 |  |
|  | 2002 | 58 | 52,999 | 1.09 | 1.23 (0.85 - 1.80) | 0.27 |  |
|  | 2003 | 30 | 52,919 | 0.57 | 0.64 (0.41 – 1.00) | 0.05 |  |
|  | 2004 | 44 | 58,778 | 0.75 | 0.84 (0.56 - 1.26) | 0.41 |  |
|  | 2005 | 40 | 60,090 | 0.67 | 0.75 (0.50 - 1.14) | 0.18 |  |
|  | 2006 | 32 | 59,242 | 0.54 | 0.61 (0.39 - 0.95) | 0.03 |  |
|  | 2007 | 42 | 60,078 | 0.70 | 0.79 (0.52 - 1.19) | 0.25 |  |
|  | 2008 | 44 | 61,998 | 0.71 | 0.80 (0.53 - 1.20) | 0.28 |  |
|  | 2009 | 51 | 61,968 | 0.82 | 0.93 (0.63 - 1.37) | 0.71 |  |
|  | 2010 | 58 | 60,816 | 0.95 | 1.08 (0.74 - 1.57) | 0.70 |  |
|  | 2011 | 38 | 59,592 | 0.64 | 0.72 (0.47 - 1.09) | 0.12 |  |
|  | 2012 | 53 | 58,593 | 0.9 | 1.02 (0.69 - 1.50) | 0.92 |  |
|  | 2013 | 51 | 57,520 | 0.89 | 1 |  |  |
|  |  |  |  |  |  |  |  |
| Season | Spring | 120 | 186,632 | 0.64 | 1 |  | 0.13 |
|  | Summer | 240 | 311,753 | 0.77 | 1.20 (0.96 - 1.49) | 0.11 |  |
|  | Autumn | 176 | 207,788 | 0.85 | 1.32 (1.04 - 1.66) | 0.02 |  |
|  | Winter | 74 | 100,591 | 0.74 | 1.14 (0.86 - 1.53) | 0.36 |  |
|  |  |  |  |  |  |  |  |
| Number of runners | 1 to 8 | 121 | 167,238 | 0.72 | 1 |  | 0.22 |
|  | 9 to 11 | 194 | 234,612 | 0.83 | 1.14 (0.91 - 1.43) | 0.25 |  |
|  | 12 to 13 | 140 | 174,764 | 0.80 | 1.11 (0.87 - 1.41) | 0.41 |  |
|  | 14+ | 155 | 230,150 | 0.67 | 0.93 (0.73 - 1.18) | 0.55 |  |
|  |  |  |  |  |  |  |  |
| Seconds per furlong (winner) | <12.3 | 124 | 195,510 | 0.63 | 1 |  | 0.001 |
|  | 12.3 to 12.76 | 131 | 207,677 | 0.63 | 0.99 (0.78 - 1.27) | 0.97 |  |
|  | 12.77 to 13.39 | 171 | 201,099 | 0.85 | 1.34 (1.06 - 1.69) | 0.01 |  |
|  | >13.40 | 184 | 202,361 | 0.91 | 1.43 (1.14 - 1.80) | 0.002 |  |
|  |  |  |  |  |  |  |  |
| Auction Race | No | 560 | 753,179 | 0.74 | 1 |  | 0.14 |
|  | Yes | 50 | 53,585 | 0.93 | 1.26 (0.94 - 1.68) | 0.12 |  |
|  |  |  |  |  |  |  |  |
| Apprentice Race | No | 581 | 778,748 | 0.75 | 1 |  | 0.1 |
|  | Yes | 29 | 28,016 | 1.04 | 1.39 (0.96 - 2.02) | 0.09 |  |
|  |  |  |  |  |  |  |  |
| Novice race | No | 608 | 801,156 | 0.76 | 1 |  | 0.22 |
|  | Yes | 2 | 5,608 | 0.36 | 0.47 (0.12 - 1.88) | 0.29 |  |
|  |  |  |  |  |  |  |  |
| Seller | No | 574 | 768,428 | 0.75 | 1 |  | 0.2 |
|  | Yes | 36 | 38,336 | 0.94 | 1.26 (0.90 - 1.76) | 0.18 |  |
|  |  |  |  |  |  |  |  |
| Horse-level | |  |  |  |  |  |  |
| Age (years) | 2 | 101 | 155,367 | 0.65 | 1 |  | 0.003 |
|  | 3 | 186 | 254,906 | 0.73 | 1.12 (0.88 - 1.43) | 0.35 |  |
|  | 4 | 98 | 154,099 | 0.64 | 0.98 (0.74 - 1.29) | 0.88 |  |
|  | 5 | 81 | 94,093 | 0.86 | 1.32 (0.99 - 1.77) | 0.06 |  |
|  | 6 | 49 | 60,662 | 0.81 | 1.24 (0.88 - 1.75) | 0.21 |  |
|  | 7+ | 95 | 87,637 | 1.08 | 1.67 (1.26 - 2.21) | <0.001 |  |
|  |  |  |  |  |  |  |  |
| First year racing | No | 413 | 583,391 | 0.71 | 1 |  | 0.01 |
|  | Yes | 197 | 223,272 | 0.88 | 1.25 (1.05 - 1.48) | 0.01 |  |
|  |  |  |  |  |  |  |  |
| Number of starts | |  |  |  | 0.99 (0.99 – 1.00) | 0.03 | 0.02 |
|  |  |  |  |  |  |  |  |
| Eye cover | No | 517 | 701,796 | 0.74 | 1 |  | 0.02 |
|  | Yes, first time | 28 | 20,654 | 1.36 | 1.84 (1.26 - 2.69) | <0.001 |  |
|  | Yes, worn previously | 65 | 84,314 | 0.77 | 1.05 (0.81 - 1.35) | 0.73 |  |
|  |  |  |  |  |  |  |  |
| Horse average performance score | | | |  | 1.01 (0.99 - 1.03) | 0.21 | 0.21 |
|  |  |  |  |  |  |  |  |
| Percentage of horse wins | 0 | 304 | 366,019 | 0.83 | 1 |  | <0.001 |
|  | <6.00 | 21 | 37,463 | 0.56 | 0.67 (0.43 - 1.05) | 0.08 |  |
|  | 6.01 to 14.29 | 122 | 205,102 | 0.59 | 0.72 (0.58 - 0.88) | 0.002 |  |
|  | >14.29 | 163 | 198,180 | 0.82 | 0.99 (0.82 - 1.20) | 0.92 |  |
|  |  |  |  |  |  |  |  |
| Percentage of horse places | 0 | 169 | 218,064 | 0.78 | 1 |  | <0.001 |
|  | <28.57 | 138 | 191,856 | 0.72 | 0.93 (0.74 - 1.16) | 0.52 |  |
|  | 28.58 to 42.86 | 123 | 202,812 | 0.61 | 0.78 (0.62 - 0.99) | 0.04 |  |
|  | >42.86 | 180 | 194,032 | 0.93 | 1.2 (0.97 - 1.48) | 0.09 |  |
|  |  |  |  |  |  |  |  |
| Percentage of horse failure to finish | None | 599 | 783,158 | 0.76 | 1 |  | 0.08 |
|  | At least one | 11 | 23,606 | 0.47 | 0.61 (0.34 - 1.11) | 0.10 |  |
|  |  |  |  |  |  |  |  |
| Trainer-level | |  |  |  |  |  |  |
| Percentage of trainer wins | | |  |  | 1.02 (1.00 - 1.03) | 0.02 | 0.03 |
|  |  |  |  |  |  |  |  |
| Percentage of prior places | | |  |  | 1.01 (1.00 - 1.02) | 0.08 | 0.09 |
|  |  |  |  |  |  |  |  |
| Percentage of prior failure to finish | <0.19 | 170 | 213,175 | 0.80 | 1 |  | 0.008 |
|  | 0.19 to 0.31 | 116 | 200,291 | 0.58 | 0.73 (0.57 - 0.92) | 0.01 |  |
|  | 0.32 to 0.47 | 162 | 198,065 | 0.82 | 1.03 (0.83 - 1.27) | 0.82 |  |
|  | >0.47 | 162 | 195,233 | 0.83 | 1.04 (0.84 - 1.29) | 0.72 |  |
|  |  |  |  |  |  |  |  |
| Jockey-level | |  |  |  |  |  |  |
| Percentage prior jockey wins | <7.19 | 146 | 201,785 | 0.72 | 1 |  | 0.23 |
|  | 7.19 to 9.22 | 153 | 202,218 | 0.76 | 1.05 (0.83 - 1.31) | 0.70 |  |
|  | 9.23 to 11.24 | 173 | 201,695 | 0.86 | 1.19 (0.95 - 1.48) | 0.13 |  |
|  | >11.24 | 138 | 201,066 | 0.69 | 0.95 (0.75 - 1.20) | 0.66 |  |
