# Supplementary material for: Risk factors for race-day fatality in flat racing Thoroughbreds in Great Britain (2000 to 2013)
Source: PLoS One. 2018 Mar 21;13(3):e0194299. doi: 10.1371/journal.pone.0194299 (PMC5862470; doi:10.1371/journal.pone.0194299)
Supplement: S2 Table — Values shown for variables with a likelihood P value of <0.25 and screened for inclusion in the multivariable model. (DOCX) [file pone.0194299.s002.docx]

S2 Table: Univariable logistic regression results of risk factors for race-day fatality on turf racecourses in British flat racing Thoroughbreds (2000 to 2013). Values shown for variables with a likelihood P value of <0.25 and screened for inclusion in the multivariable model.

| Variable | Level | No. of Cases | No. of Starts | Incidence /1000 starts | Odds ratio (95% Confidence Interval) | Wald P value | Likelihood ratio P value |
| --- | --- | --- | --- | --- | --- | --- | --- |
| Course-level | |  |  |  |  |  |  |
| Going | Hard or firm | 24 | 29002 | 0.83 | 1.24 (0.79 - 1.93) | 0.35 | 0.04 |
|  | Good to firm | 172 | 217424 | 0.79 | 1.18 (0.93 - 1.51) | 0.18 |  |
|  | Good to firm | 102 | 152511 | 0.67 | 1 |  |  |
|  | Good to soft | 41 | 76665 | 0.53 | 0.80 (0.56 - 1.15) | 0.23 |  |
|  | Soft or heavy | 38 | 72969 | 0.52 | 0.78 (0.54 - 1.13) | 0.19 |  |
|  |  |  |  |  |  |  |  |
| Race-level | |  |  |  |  |  |  |
| Distance (metres) | |  |  |  | 1.06 (1.04 - 1.08) | <0.001 | <0.001 |
|  |  |  |  |  |  |  |  |
| Winning speed (second per furlong) (n=544,708 starts) | <12.3 | 83 | 135,900 | 0.61 | 1 |  | 0.09 |
|  | 12.3 to 12.77 | 82 | 137,645 | 0.60 | 0.98 (0.72 - 1.32) | 0.87 |  |
|  | 12.78 to 13.4 | 103 | 136,563 | 0.75 | 1.24 (0.92 - 1.65) | 0.15 |  |
|  | 13.41+ | 109 | 134,600 | 0.81 | 1.33 (1.00 - 1.76) | 0.05 |  |
|  |  |  |  |  |  |  |  |
| Season | Spring | 75 | 133,256 | 0.56 | 1 |  | 0.12 |
|  | Summer | 201 | 280,149 | 0.72 | 1.33 (0.98 - 1.79) | 0.06 |  |
|  | Autumn | 101 | 135,166 | 0.75 | 1.27 (0.98 - 1.66) | 0.07 |  |
|  |  |  |  |  |  |  |  |
| Flat season | Off (Mar/Nov) | 3 | 13,655 | 0.22 | 1 |  | 0.04 |
|  | Shoulder (Apr/Oct) | 61 | 95,146 | 0.64 | 2.92 (0.92 - 9.31) | 0.07 |  |
|  | On | 313 | 439,770 | 0.71 | 3.24 (1.04 - 10.11) | 0.04 |  |
|  |  |  |  |  |  |  |  |
| Month | March | 2 | 6,432 | 0.31 | 1 |  | 0.05 |
|  | April | 17 | 41,921 | 0.41 | 1.30 (0.30 - 5.65) | 0.72 |  |
|  | May | 56 | 84,903 | 0.66 | 2.12 (0.52 - 8.7) | 0.30 |  |
|  | June | 70 | 93,804 | 0.75 | 2.40 (0.59 - 9.79) | 0.22 |  |
|  | July | 60 | 92,754 | 0.65 | 2.08 (0.51 - 8.52) | 0.31 |  |
|  | August | 71 | 93,591 | 0.76 | 2.44 (0.60 - 9.95) | 0.21 |  |
|  | September | 56 | 74,718 | 0.75 | 2.41 (0.59 - 9.88) | 0.22 |  |
|  | October | 44 | 53,225 | 0.83 | 2.66 (0.64 - 10.97) | 0.18 |  |
|  | November | 1 | 7,223 | 0.14 | 0.45 (0.04 - 4.91) | 0.51 |  |
|  |  |  |  |  |  |  |  |
| Group 1 race | No | 366 | 543,966 | 0.67 | 1 |  | <0.001 |
|  | Yes | 11 | 4,605 | 2.39 | 3.56 (1.95 - 6.48) | <0.001 |  |
|  |  |  |  |  |  |  |  |
| Handicap race | No | 213 | 329,582 | 0.65 | 1 |  | 0.16 |
|  | Yes | 164 | 218,989 | 0.75 | 0.86 (0.70 - 1.06) | 0.16 |  |
|  |  |  |  |  |  |  |  |
| Rated race | No | 250 | 345,841 | 0.72 | 1 |  | 0.19 |
|  | Yes | 127 | 202,730 | 0.63 | 0.87 (0.70 - 1.07) | 0.19 |  |
|  |  |  |  |  |  |  |  |
| Conditional race | No | 372 | 536,633 | 0.69 | 1 |  | 0.22 |
|  | Yes | 5 | 11,938 | 0.42 | 0.60 (0.25 - 1.46) | 0.26 |  |
|  |  |  |  |  |  |  |  |
| Horse-level | |  |  |  |  |  |  |
| Age (years) | 2 | 72 | 119,631 | 0.60 | 1 |  | 0.02 |
|  | 3 | 129 | 181,290 | 0.71 | 1.18 (0.89 - 1.58) | 0.26 |  |
|  | 4 | 49 | 99,277 | 0.49 | 0.82 (0.57 - 1.18) | 0.28 |  |
|  | 5 | 49 | 58,347 | 0.84 | 1.40 (0.97 - 2.01) | 0.07 |  |
|  | 6 | 30 | 37,453 | 0.80 | 1.33 (0.87 - 2.04) | 0.19 |  |
|  | 7+ | 48 | 52,573 | 0.91 | 1.52 (1.05 - 2.19) | 0.03 |  |
|  |  |  |  |  |  |  |  |
| First year racing | No | 238 | 382,847 | 0.62 | 1 |  | 0.01 |
|  | Yes | 139 | 165,724 | 0.84 | 1.35 (1.09 - 1.66) | 0.01 |  |
|  |  |  |  |  |  |  |  |
| Number of starts (turf) | | |  |  | 0.99 (0.98 - 0.998) | 0.02 | 0.01 |
|  |  |  |  |  |  |  |  |
| Days since last race (racing intensity) | First race | 44 | 62,859 | 0.70 | 1 |  | 0.02 |
|  | 1 to 7 | 27 | 47,064 | 0.57 | 0.82 (0.51 - 1.32) | 0.42 |  |
|  | 8 to 93 | 252 | 364,720 | 0.69 | 0.99 (0.72 - 1.36) | 0.94 |  |
|  | 94 to 364 | 40 | 66,085 | 0.61 | 0.86 (0.56 - 1.33) | 0.51 |  |
|  | 365+ | 14 | 7,843 | 1.79 | 2.55 (1.40 - 4.66) | <0.001 |  |
|  |  |  |  |  |  |  |  |
| 365 days or more since last race start | No | 363 | 540,365 | 0.67 | 1 |  | 0.002 |
|  | Yes | 14 | 7,829 | 1.79 | 1.79 (1.56 - 4.54) | <0.001 |  |
|  |  |  |  |  |  |  |  |
| Eye cover | No | 326 | 490,679 | 0.66 | 1 |  | 0.02 |
|  | Yes, first time | 21 | 19,676 | 1.07 | 1.07 (1.32 - 3.20) | 0.001 |  |
|  | Yes, worn previously | 30 | 38,216 | 0.79 | 0.79 (0.73 - 1.54) | 0.75 |  |
|  |  |  |  |  |  |  |  |
| Horse average performance score (all flat racing starts) | | | |  | 1.01 (0.99 - 1.03) | 0.25 | 0.24 |
|  |  |  |  |  |  |  |  |
| Percentage of horse wins | 0 | 211 | 286005 | 0.74 | 1 |  | 0.01 |
|  | 0.01 to 14.3 | 64 | 126531 | 0.51 | 0.69 (0.52 - 0.91) | 0.01 |  |
|  | >14.3 | 102 | 136035 | 0.75 | 1.02 (0.80 - 1.29) | 0.89 |  |
|  |  |  |  |  |  |  |  |
| Percentage of horse places | 0 | 128 | 179,417 | 0.71 | 1 |  | 0.005 |
|  | 0.01 to 26.7 | 61 | 95,225 | 0.64 | 0.90 (0.66 - 1.22) | 0.49 |  |
|  | 26.8 to 43.7 | 70 | 137,025 | 0.51 | 0.72 (0.53 - 0.96) | 0.03 |  |
|  | >43.7 | 118 | 136,904 | 0.86 | 1.21 (0.94 - 1.55) | 0.14 |  |
|  |  |  |  |  |  |  |  |
| Percentage of horse failure to finish | No | 373 | 537653 | 0.69 | 1 |  | 0.16 |
|  | Yes | 4 | 10918 | 0.37 | 0.53 (0.20 - 1.41) | 0.2 |  |
|  |  |  |  |  |  |  |  |
| Trainer-level | |  |  |  |  |  |  |
| Trainer average performance (turf) | <12.8 | 99 | 138515 | 0.71 | 1 |  | 0.04 |
|  | 12.8 to 13.4 | 75 | 136511 | 0.55 | 0.77 (0.57 - 1.04) | 0.09 |  |
|  | 13.5 to 14.4 | 89 | 137029 | 0.65 | 0.91 (0.68 - 1.21) | 0.51 |  |
|  | >14.4 | 114 | 136516 | 0.84 | 1.17 (0.89 - 1.53) | 0.26 |  |
|  |  |  |  |  |  |  |  |
| Percentage of trainer wins (turf) | | | |  | 1.02 (1 - 1.04) | 0.02 | 0.02 |
|  |  |  |  |  |  |  |  |
| Percentage of prior places (turf) | <21.8 | 100 | 137205 | 0.73 | 1 |  | 0.002 |
|  | 21.8 to 26.5 | 65 | 137297 | 0.47 | 0.65 (0.48 - 0.89) | 0.01 |  |
|  | 26.6 to 33.2 | 98 | 137179 | 0.71 | 0.98 (0.74 - 1.30) | 0.89 |  |
|  | >33.2 | 114 | 136890 | 0.83 | 1.14 (0.87 - 1.49) | 0.33 |  |
|  |  |  |  |  |  |  |  |
| Percentage of prior failure to finish (turf) | <0.14 | 100 | 137628 | 0.73 | 1 |  | 0.02 |
|  | 0.14 to 0.27 | 71 | 137180 | 0.52 | 0.71 (0.53 - 0.97) | 0.03 |  |
|  | 0.28 to 0.45 | 112 | 136952 | 0.82 | 1.13 (0.86 - 1.47) | 0.39 |  |
|  | >0.45 | 94 | 136811 | 0.69 | 0.95 (0.71 - 1.25) | 0.70 |  |
|  |  |  |  |  |  |  |  |
| Jockey-level | |  |  |  |  |  |  |
| Jockey average performance (turf) | | | |  | 1.06 (0.98 - 1.13) | 0.13 | 0.12 |
